# Psychological Outcomes in Children and Early Adolescents With Type 1 Diabetes Following Pediatric Diabetes Summer Camp: A 3-Month Follow-Up Study

**DOI:** 10.3389/fped.2021.650201

**Published:** 2021-03-10

**Authors:** Alda Troncone, Antonietta Chianese, Crescenzo Cascella, Angela Zanfardino, Dario Iafusco

**Affiliations:** ^1^Department of Psychology, University of Campania “L. Vanvitelli”, Caserta, Italy; ^2^Department of the Woman, Child and General and Specialized Surgery, University of Campania “L. Vanvitelli”, Naples, Italy

**Keywords:** type 1 diabetes, adolescence, children, summer camp, psychological adjustment, illness perception, diabetes burden, treatment satisfaction

## Abstract

**Objective:** The aim of this study was to assess general psychosocial adjustment to diabetes and perceived disease management among patients with type 1 diabetes (T1D) and their parents before and after patients' participation in a diabetes summer camp.

**Methods:** In this follow-up study, 20 children and adolescents with T1D (eight boys; mean age = 11.01 ± 0.94 years; mean diabetes duration = 3.02 ± 2.27) attending a southern Italian diabetic center, along with their parents, were assessed prior to and 3 months after the youths participated in a 1 week camp-based intervention involving didactic and interactive child-centered education and recreational activities. Patients and their parents completed measures assessing patients' quality of life and strategies employed by patients to cope with pain. Patients also completed measures evaluating their diabetes psychosocial adjustment, diabetes self-efficacy management, and illness perception; also, their parents completed measures of caregivers' perceived diabetes burden and treatment satisfaction. Youths' glycated hemoglobin (HbA1c) and standardized body mass index (z-BMI) values were also assessed. Within-subjects repeated-measures analyses of variance evaluated pre- and post-camp changes.

**Results:** Camp attendance showed no beneficial effects on glycemic control, as indicated by HbA1c values both before (7.02%) and after (7.28%) camp being lower than 7.5%. HbA1c values were found to have increased after camp (pre-camp = 7.02%, post-camp = 7.28%; *p* = 0.010), but since they still fell within an acceptable range, they did not reveal clinically relevant changes in glycemic control. No substantial significant improvement in psychosocial measures was observed in children or parents (all *p* > 0.05). According to the parents' evaluation, social support-seeking as a patient pain-coping strategy was slightly increased (*p* = 0.044) after attending the camp.

**Conclusions:** This study does not provide empirical evidence of benefits of participating in a diabetes camp for either patients or their parents. These findings suggest that healthcare providers rethink such camps as an experience for youths with T1D that actively involves parents and that includes both youth- and parent-focused psychological interventions.

## Introduction

The psychological burden imposed by type 1 diabetes (T1D) is taxing. Due to the characteristics of the disease, children and adolescents with T1D must monitor and fulfill complex health needs, such as blood glucose monitoring, insulin therapy, and dietary restrictions and planning. Therefore, they are required to change their everyday life in different ways, and all these behavioral challenges place serious demands upon the youth and their family, negatively impacting individual psychological functioning. Unsurprisingly, youths with T1D have frequently been described as being at high risk for psychological symptoms (1). In particular, numerous studies have indicated that individuals with T1D have a greater incidence of depression and anxiety (2, 3), diabetes distress (4), body image concerns (5, 6), and disordered eating behaviors (7) than their peers without diabetes.

Diabetes camp has been deemed worldwide as a part of diabetes care, an opportunity to offer diabetes education to children and adolescents in a group in a safe environment. As underscored in ADA recommendations (8), the mission of specialized camps for children and adolescents with diabetes is to enable youth with diabetes to learn to be more responsible for their condition within a context where they can meet and share their experiences with others and where they can have a safe, integrated educational experience. Camp may be an opportunity for children with T1D to gain or improve the ability to actively manage their illness by enhancing self-management skills (8, 9). In addition, by providing opportunities for children to participate in typical childhood and adolescent activities in a safe and inclusive space, the summer camp may meet children's psychological needs, thereby helping to develop self-confidence and supporting their overall development.

Several studies have examined the psychological effects of participation in a diabetes camp on youth with T1D. In particular, as highlighted by Anarte et al. (10), in terms of the psychological outcomes, the majority of studies has investigated children's quality of life, attitude toward illness, self-efficacy related to disease management, and concepts such as anxiety, affectivity, knowledge about disease management, adaptation, self-esteem, self-reported adherence, and so on.

While general agreement exists on the effectiveness of camp experiences in improving diabetes knowledge and management (11, 12), contradictory results are reported on psychological outcomes. While literature reviews have indicated a general improvement in psychological variables after attending a diabetes camp (13–15), especially in short-term benefits (16), other studies have described no relevant variations in anxiety and psychological adaptation after diabetes camp (17, 18). In the same way, in research investigating children's quality of life after the camp, some studies have found improvement in this dimension (10, 19) while other studies have not (14, 20–22).

Similarly, improvements in attitude toward illness, self-efficacy, competence in diabetes management, adherence, and self-care after the camp experience have been described (12, 17, 22–25), along with other evidence, all indicating mixed results (26).

In addition, to date, some psychological aspects seem to be overlooked in this research area. It is well-known that multiple-injection therapy can induce discomfort and distress to such an extent that fear of injections and finger pricks appear to be fairly common in children and adolescents with T1D (27–30) and significantly correlated with higher injection pain levels (31, 32), regardless of needle diameter (33). Pain associated with insulin injection was found in turn to severely impact the ability to self-manage diabetes, inducing patients to avoid or reduce blood glucose monitoring and insulin injections and thus worsening adherence to insulin therapy (31, 34). Despite how pain affects diabetes care, to our knowledge, no studies have been carried out researching camp's effect on youths' strategies to cope with pain as a dimension of diabetes management.

Furthermore, it should be noted that within the literature evaluating psychological outcomes of diabetes camp experiences, some studies have focused on the effects of participation on campers' parents. In particular, the majority of this research has focused on parents' satisfaction with camp experience (24, 26), on what was changed in their child (in terms of patients' adherence, self-care skills, diabetes knowledge, and management) according to the parents' point of view (12, 21, 25), and on parents' reports of what their child needed to learn (23). However, despite the positive association between parents' well-being and children's metabolic control (35), little research has investigated changes in parents' diabetes treatment-related burden or in parents' feeling and stress around managing a chronic illness after their children attended the camp (10, 22, 36). No evidence has been provided on changes in parents' treatment satisfaction.

In light of the conflicting results in this research area and of the overlooking of certain psychological aspects by previous studies, the present study sought to further investigate the psychological outcomes of patients and their caregivers after the youths participated in a 1-week diabetes camp.

In particular, the psychological benefits evaluated in youths were quality of life, strategies employed by youths to cope with pain, youths' adjustment to and perception of diabetes, and youths' confidence in self-care management of their diabetes.

Some evidence suggests that children and adolescents with T1D may wish to avoid revealing their problems (37), and some evidence indicates that they sometimes report fewer behavioral problems or diverge from their parents' reports regarding their diabetes (38, 39) or aspects of health-related quality of life (13). Thus, where measures were available (i.e., quality of life, strategies to cope with pain), parent-report evaluation was also carried out as an appropriate strategy to enable more accurately analyzing psychological variables under examination.

Additionally, in light of conflicting results on glycemic control changes after camp experiences—such that participation in diabetes camp has been described as either having a positive effect (12, 36, 40) or having no effect (21, 41) on glycemic control—changes in diabetes control, as indicated by glycated hemoglobin (HbA1c) values, were also investigated. Finally, since body mass index (BMI) is commonly considered as a nutritional status and a general health indicator of overall health (42, 43), BMI changes after camp were also evaluated.

In sum, the specific aims of this study were to assess:

(1) Campers' psychological benefits after attending the diabetes camp;
(2) Parents' changes in burden perception and treatment satisfaction following their children's camp experience.

We hypothesized that after the camp experience, changes in general psychological adjustment to diabetes and in related management would be observed in both youths and their caregivers.

## Materials and Methods

### Participants

During the period January–February 2018, youths aged 10–12 years (and their parents) attending a southern Italian diabetic center, who had never gone to a summer camp and who were using multiple daily injections (MDI), were approached during a routine clinic visit and offered to participate in a week-long overnight summer camp for children with T1D. Camp was exclusively proposed to youths treated with MDI because, as is highlighted by results from systematic reviews (44, 45), this treatment is more frequently associated with poorer glycemic control in comparison to continuous subcutaneous insulin infusion. The first 20 parents/youths who agreed and were registered to attend the summer camp were eligible for participation. Parent–youth dyads who were accepted to attend the camp were offered enrollment in the study.

Inclusion criteria for the study included (a) child camper with a diagnosis of T1D for at least 1 year (to avoid the “honeymoon period” and to allow families time to adjust to the diagnosis) and (b) child and primary caregiver present for questionnaire completion, who were able to read and understand the questionnaires. No prespecified HbA1c requirement or insulin administration mode method was set for eligibility. Exclusion criteria included having other illnesses (severe disability due to disease, significant comorbidity, other diagnosed diseases) and presence of recent life stressors. A systematic examination of participants' clinical records was conducted to ascertain that the inclusion/exclusion criteria were met. Demographic and clinical data of participants are shown in Table 1.

**Table 1:** Demographic and clinical data of patients with type 1 diabetes.

	**Type 1 diabetes**
Sample size (*N*)	20
Gender (*N*) (male/female)	8/12
Age (years)	11.01 (0.94)
HbA1c (%)	7.02 (0.77)
Diabetes duration (years)	3.02 (2.27)
z-BMI	0.15 (1.08)

*Data are presented as mean values and standard deviations unless otherwise stated*.

*N, number of subjects; HbA1c, glycated hemoglobin; z-BMI, standardized body mass index*.

### Measures

#### Sociodemographic and Clinical Data

A brief interview schedule was specifically designed and completed by the clinicians to record the demographic and clinical data, including the youth's date of diagnosis, age, sex, height, weight, current HbA1c values, and other medical conditions. Possible missing data were obtained by reviewing their medical chart.

#### Psychosocial/Psychological Measures

##### Youths

*Quality of Life.* Youths' quality of life was assessed with the Pediatric Quality of Life Inventory 3.0 Type 1 Diabetes (PedsQL 3.0 DM) self- and parent-report modules (46). The PedsQL 3.0 DM is composed of 28 items across five scales: diabetes symptoms (e.g., I have to go to the bathroom too often, I feel tired or fatigued); treatment barriers (e.g., I am embarrassed about having diabetes, It is hard for me to stick to my diabetes care plan); treatment adherence (e.g., It is hard for me to take blood glucose tests, It is hard to me to exercise); worry (e.g., I worry about “going low,” I worry about long-term complication from diabetes); and communication (e.g., It is hard form me to tell the doctors and nurses how I feel, It is hard for me to ask the doctors and nurses questions). Higher scores indicate fewer problems and better quality of life. Validity and reliability studies of the PedsQL 3.0 DM have been conducted in many countries and have indicated satisfactory psychometric properties (47–49). The present study adopted the Italian translation of the PedsQL (8–12 years version), which has demonstrated good validity and reliability (50) and has been used in previous studies (51, 52).

*Coping Strategies for Physical Pain.* The Waldron/Varni Pediatric Pain Coping Inventory (PPCI) (53) is a self-report questionnaire designed to measure children's strategies of coping with physical pain. It includes both patient and parent-proxy reports and contains items asking to rate how frequently the child uses each coping skill. The PPCI is composed of five scales: cognitive self-instruction (e.g., pretend I do not have any pain or hurt), seeks social support (e.g., tell my mother or father), problem solving (e.g., ask for medicine), distraction (e.g., try not to think about the pain or hurt or ignore the pain or hurt), and catastrophizing/helplessness (e.g., yell or cry). Higher scores on all subscales indicate more adaptive coping with pain. Both versions of the questionnaire (for children and for parents) have shown good validity and reliability (54–56). A validated 24-item Italian version of the PPCI was used in this study (57).

*Psychological Adjustment to Diabetes.* The Diabetes Attitude Questionnaire (ATT19) (58) is a 19-item self-report questionnaire designed to assess the emotional adjustment to diabetes and to evaluate the extent to which diabetes is integrated into the patient's lifestyle and personality (e.g., I dislike to be referred as a diabetic; Most people would find it difficult to have diabetes). Higher scores indicate that patients are more likely to be well-adjusted to their chronic illness. A number of studies have examined the ATT's psychometric characteristics and have shown sound reliability and validity (59–61), including an Italian validation study (62).

*Perception of Illness.* The Brief Illness Perception Questionnaire (Brief IPQ) (63) is a self-report questionnaire composed of eight items aimed at assessing children's cognitive representation (e.g., How long do you think your illness will continue?) and emotional representation (e.g., How concerned are you about your illness?) of their illness, as well as their illness comprehensibility (e.g., How well do you feel you understand your illness?). Higher scores suggest stronger perceptions along that dimension. Several studies have demonstrated that the Brief IPQ has good reliability (64) and overall good psychometric properties (65, 66). A validated Italian version of the Brief IPQ was used in this study (67).

*Self-Efficacy in Diabetes Management.* The Diabetes Management Self-Efficacy Scale (DMSES) (68) is a 15-item self-report measure that assesses the individual's confidence in self-management of diabetes activities (e.g., to what extent do you feel able to “keep my weight under control;” “adjust my diet when increasing exercise”). Higher scores indicate higher levels of perceived self-efficacy. The DMSES has been validated in several languages and countries, demonstrating acceptable reliability and validity (69–71). A validated Italian version of the DMSES was used in the present study (72).

##### Parents

*Diabetes Treatment Satisfaction.* The Diabetes Treatment Satisfaction Questionnaire for parents (DTSQ-parent) (73) is a parent-report measure designed to assess parents' satisfaction with the current treatment of their children. It consists of 14 items concerning general diabetes treatment satisfaction (e.g., How satisfied are you with your child's current treatment?, How easy or difficult is your child's diabetes treatment?), the perceived frequencies of hypoglycemia (e.g., How often have you felt that your child's blood sugars have been too low lately?), and perceived diabetes control and effects on parents' lives (e.g., How well-controlled do you feel your child's diabetes has been lately?). A higher score indicates greater satisfaction. The DTSQ is used internationally to measure treatment satisfaction and has been proven to have good psychometric properties, including parent version (73–75).

*Perceived Burden.* The Problem Areas in Diabetes parent revised version questionnaire (PAID-PR) (76) is an 18-item measure of the perceived parental burden associated with caring for a child with diabetes (e.g., I feel “burned out” by the constant effort to manage diabetes). Higher scores indicate greater perceived distress and more parental burden. Studies on the psychometric properties of PAID-PR have shown good internal consistency, test–retest reliability, and concurrent validity (76–78). The present study adopted a validated Italian version of the PAID-PR that has also been used in previous Italian studies (52, 79).

### Camp Setting

The summer camp was located in a seaside resort on the Cilento coast in southern Italy. It lasted 7 days and was supported by a contribution from a public fund. The facility was adequately equipped for camp purposes, with large rooms for educational activities to be held in groups and suitable space for sports and recreational activities (e.g., a beach).

In line with ADA recommendations (8), the medical staff was composed of one medical director (a physician with expertise in managing type 1 diabetes), one physician with an interest in diabetes, one medical resident, one dietitian with expertise in diabetes, and one psychologist. All staff were previously appropriately trained about routine diabetes management, issues related to lifestyle modification for T1D, signs and symptoms of hypo-/hyperglycemia, and the treatment of diabetes-related emergencies.

The day was organized as follows. About 1 h a day was planned after breakfast for specific educational activities and practical training in groups, designed to extend previous diabetes knowledge and to reinforce self control and self-management skills. Education sessions were directed by a physician (assisted by the medical resident) and by the psychologist and were held in traditional method (e.g., using slides, short films, illustrated handbooks, etc.). These activities consisted of interactive lectures and subsequent group discussion seminars about disease etiology and symptoms; insulin therapy and blood glucose monitoring; diet (carbohydrate measurements); recognition and management of hypoglycemia/hyperglycemia; the relationship connecting exercise, food intake, and insulin doses; the importance of diabetes control; disease evolution control; daily problems related to T1D management; difficulties in living with T1D; and stress management. All content was appropriately adjusted to the age of participants. The remaining hours of the morning and at least 2 h in the afternoon were devoted to recreational activities like going to the beach and sports (volleyball, soccer, etc.). After dinner, all youths participated in leisure activities planned by the facility staff (exhibitions, dances, music, etc.).

Before each meal (breakfast, lunch, dinner, pre-evening snack, and midnight), blood glucose levels were analyzed, and insulin doses were calculated by a member of the medical staff in collaboration with the child and adapted to that day's meals on the basis of the previous day's values. Values were achieved by finger-prick blood sample tests. Additional blood glucose measurements were made if the youth reported symptoms ascribable to hypoglycemia. The nutritionist planned the diet for all participants according to their physical requirements, and it could be modified on the basis of their caloric intake daily needs.

Campers attended the camp program free of charge.

### Study Design and Procedure

This study was a follow-up investigation. All participants signed an informed consent form before participating in any study-related activities. The study was approved by the local Ethics Committee. Participation was voluntary, and no incentives were offered.

The youth and their primary caregiver were seen at baseline (T0) and at 3 months (T1) after the camp. The primary caregiver was identified as the person who is most responsible for the daily care of the youth with T1D. Two weeks prior to the camp session, all registered campers were met at the clinic in order to inform them about the camp and to have the informed consent form signed by the parents (children provided assent to participate in the study) (baseline, T0). After clinicians gathered demographic and medical data, interviews and test administrations were conducted by a psychologist with a bachelor's degree in Psychology who was adequately trained in the techniques and who had prior experience with the instruments. Evaluations were made individually and anonymously in a quiet, comfortable room made available by the clinic. The order of questionnaire administration was randomly assigned. At T1, which was planned during a routine clinic visit, HbA1c measurements and questionnaire completion were conducted following the same procedure.

### Statistical Analysis

Cronbach's alpha (α) was used to assess the homogeneity of the scales. Comparisons of means at two different time points (baseline–T1) were conducted separately for patients and parents using repeated-measures analyses of variance (ANOVA). Scores of changes were computed for HbA1c, BMI, and psychosocial measures. Results were considered significant at alpha = 0.05 for a two-sided test. Effect size was reported as partial Eta square. The statistical analysis was performed with Statistical Package for the Social Sciences (SPSS) version 21.0 for Macintosh.

## Results

### Sample Characteristics

Of the 20 camp attendees, 20 parent–youth dyads agreed to participate in the study. Twenty youths (eight boys) and their caregivers (one father) consented to the study and completed the 3-month post-camp follow-up study. One parent (mother) was excluded after failing to complete the full evaluation (post-test).

Table 1 presents the gender, age, duration of illness, HbA1c, BMI, and standardized BMI (z-BMI) of the participants.

### Glycemic Control and z-BMI

As shown in Table 2, no significant clinical improvements were found in HbA1c values from before camp to after camp; HbA1c values were even found to increase after camp (*p* = 0.010). No significant differences were found in z-BMI values (*p* = 0.085).

**Table 2:** Means (*SD*) for Hb1Ac, z-BMI, PedsQL, PPCI, ATT19, DMSES, and B-IPQ for participants (*N* = 20) at each time point.

	**Baseline (T0)**	**3 months (T1)**	**Overall changes Repeated measures ANOVA**		
**Outcomes**	***M* (*SD*)**	***M* (*SD*)**	***F***	***p***	***n*^**2**^**
HbA1c	7.02 (0.77)	7.28 (0.84)	8.172	0.010	0.301
z-BMI	0.15 (1.08)	0.27 (1.05)	3.291	0.085	–
**PedsQL self-report**					
Diabetes symptoms	68.83 (14.11)	62.73 (12.84)	3.700	0.070	–
Treatment barriers	82.18 (16.75)	79.06 (20.41)	0.455	0.508	–
Treatment adherence	84.82 (15.75)	83.57 (17.93)	0.082	0.777	–
Worry	60.83 (24.65)	58.75 (24.55)	0.126	0.726	–
Communication	77.92 (24.82)	79.58 (20.32)	0.065	0.801	–
**PPCI**					
Cognitive self-instruction	3.8 (2.17)	3.65 (2.3)	0.051	0.823	.
Problem-solving	4.7 (2.62)	3.8 (2.55)	1.856	0.189	–
Distraction	6.9 (3.51)	7.4 (3.22)	0.492	0.491	–
Seek social support	5.7 (2.83)	5.45 (2.84)	0.216	0.647	–
Catastrophizing/helplessness	4.3 (1.69)	3.6 (1.31)	2.160	0.158	.
**ATT19**	61.2 (19.96)	66.2 (12.5)	2.408	0.137	–
**DMSES**					
Disease management	7.54 (1.63)	7.64 (1.23)	0.039	0.845	–
Lifestyle management	6.98 (2.1)	7.22 (1.58)	0.257	0.618	
**B-IPQ**					
Cognitive representations	37.21 (3.71)	37.63 (4.36)	0.302	0.589	
Emotional representations	12.26 (5.24)	12.89 (3.62)	0.540	0.472	
Comprehensibility	8.00 (2.75)	7.74 (1.69)	0.195	0.664	

*Data are presented as mean values and standard deviations unless otherwise stated*.

*HbA1c, glycated hemoglobin; z-BMI, standardized body mass index; PedsQL, Pediatric Quality of Life Inventory; PPCI, Pediatric Pain Coping Inventory; ATT19, Diabetes Attitude Questionnaire; DMSES, Diabetes Management Self-Efficacy Scale; B-IPQ, Brief Illness Perception Questionnaire*.

### Psychological Outcomes

Cronbach's alpha for all adopted measures (total score) demonstrated adequate internal consistency (PedsQL child self-report α = 0.873; ATT19 α = 0.886; PPCI–patient proxy report α =0.852; DMSES α = 0.899; PedsQL parent proxy report α = 0.848; PAID-PR α = 0.779; PPCI–parent proxy report α = 0.738; DTSQ α = 0. 829) except for Brief IPQ (α = 0.338).

#### Youths

The first 3 months after camp, the youths' quality of life (according to youths' and parents' opinions) was not found to be significantly changed, as measured by the PedsQL 3.0 DM's subscales scores (Diabetes symptoms, Treatment barriers, Treatment adherence, Worry, Communication, all *p* > 0.05). From T0 to T1, strategies employed by youths to cope with pain (according to youths' and parents' perspectives) were found not substantially changed, as measured by PPCI's subscales (Cognitive self-instruction, Problem solving, Distraction, Social support seeking, Catastrophizing/helplessness, all *p* > 0.05). According to parents' evaluations, social support seeking as a pain coping strategy was slightly increased (*p* = 0.044) after their child participated in the camp.

Similarly, patients did not report significant improvements in their adjustment to diabetes (ATT19 scores *p* > 0.05), confidence in self-care management of their diabetes (Disease management and Lifestyle management DMSES subscales, *p* > 0.05), or perception of disease (as Illness Cognitive/Emotional Representations and Comprehensibility IPQ scores subscales, *p* > 0.05).

In Table 2, the mean values and effect sizes for HbA1c, z-BMI, and psychological measures for participants at each time point are shown.

#### Parents

From T0 to T1, all parents' psychological scores remained stable. In particular, after their child participated in the camp, parents did not report significant improvement in diabetes burden perception or in treatment satisfaction, according to PAID-PR scores and DTSQ subscales scores, respectively (general Treatment satisfaction, Perceived diabetes control, Perceived hypoglycemia, all *p* > 0.05).

In Table 3, the mean values and effect sizes of the psychological measures for participants' parents at each time point are shown.

**Table 3:** Means (*SD*) for PedsQL, PPCI, PAID-PR, DTSQ for participants' parents (*N* = 20) at each time point.

	**Baseline (T0)**	**3 months (T1)**	**Overall changes Repeated measures ANOVA**		
**Outcomes**	***M* (*SD*)**	***M* (*SD*)**	***F***	***p***	***n*^**2**^**
**PedsQL parents' version**					
Diabetes symptoms	63.16 (15.07)	65.6 (12.99)	0.822	0.377	–
Treatment barriers	68.42 (18.57)	64.8 (19.23)	0.397	0.536	–
Treatment adherence	74.47 (16.64)	77.76 (18.09)	0.573	0.459	–
Worry	44.74 (24.72)	37.72 (20.29)	1.209	0.286	–
Communication	79.38 (20.85)	73.25 (20.52)	0.939	0.345	–
**PPCI**					
Cognitive self-instruction	2.55 (1.54)	3.2 (1.15)	2.097	0.164	.
Problem-solving	4.7 (2.54)	5.65 (2.18)	3.537	0.075	–
Distraction	7.4 (3.86)	7.6 (3.59)	0.025	0.877	–
Seek social support	5.8 (2.14)	6.8 (1.64)	4.634	0.044	0.196
Catastrophizing/helplessness	3.95 (1.85)	4.4 (1.31)	1.222	0.283	–
**PAID-PR**	33.00 (14.49)	35.53 (12.46)	0.350	0.562	.
**DTSQ**					
Treatment satisfaction	42.58 (12.87)	42.11 (9.68)	0.023	0.880	.
Perceived diabetes control	6.95 (2.32)	6.63 (1.86)	0.178	0.678	
Perceived hypoglycemia	2.89 (1.61)	3.11 (1.23)	0.186	0.671	

*Data are presented as mean values and standard deviations unless otherwise stated*.

*PedsQL, Pediatric Quality of Life Inventory; PPCI, Pediatric Pain Coping Inventory; PAID-PR, Problem Areas in Diabetes parent revised; DTSQ, Diabetes Treatment Satisfaction Questionnaire*.

## Discussion

This study aimed to investigate the psychological outcomes for patients and their caregivers after the youths participated in a 1-week diabetes camp. It seeks to contribute to the scientific debate about the utility of diabetes camp in improving youths' adaptation to their illness.

Contrary to our hypothesis, the present findings showed that the camp experience was not associated with significant changes in youths' general psychological adjustment to diabetes or their perceived disease management, or in parents' overall distress regarding their child's diabetes management. In particular, as already found in previous research (14, 20–22), after youths attended summer camp, they did not report an improvement in quality of life nor were parents found to perceive their child's quality of life differently. Similarly, contrary to some previous studies (12, 17, 22–25) but consistent with other evidence indicating mixed results (26), youths did not report improvements in their ability to manage their diabetes, in their adjustment to illness, or in their diabetes perception after the camp experience.

In a comparison of pre-camp and post-camp values, a significant change following camp attendance was only found in youths' strategies to cope with pain according to the parents' perception. Specifically, after the camp, parents reported that their child had an increased tendency to cope with pain by seeking social support. Nevertheless, it should be noted that this finding was at borderline significance; therefore, this improvement cannot be considered a relevant change in general coping pain strategies adopted by youths after diabetes camp.

In terms of parents' perceived burden and treatment satisfaction, no significant changes were detected. It should be noted that few studies have explored and supported the beneficial effect of diabetes camp on parents' burden (22, 36), and no publications exist on longitudinal changes in parents' treatment satisfaction as an outcome of camp experience. As such, this is the first study evaluating this psychological dimension of parents, and it is potentially relevant to stimulating further research in this area.

In addition, HbA1c values were found to have actually increased after diabetes camp—albeit in an acceptable range, analogous to population data in similar age groups (80, 81)—supporting previous evidence that did not reveal any improvement in glycemic control after camp experience (21, 41).

Overall, this study does not provide empirical evidence of the benefits of participation in a diabetes camp, either in campers or in their parents, and it could be included among the studies in this research area reporting conflicting results about psychological positive outcomes of camp. In this regard, it should be noted that some authors have highlighted that an overall conclusion on the psychological outcomes of this experience is difficult to reach, due to huge variations in the methodological approach, the characteristics of study populations, and the definition of camp programs (15). Several methodological limitations (e.g., the lack of a control group or longer-term follow-up measures in most studies) prevent drawing robust conclusions about the positive impact on youths' psychosocial functioning and health (16).

However, the lack of improvement in children's general diabetes adjustment and perceived management as well as in parents' perceived burden and treatment satisfaction, as found in the present study, lead us to consider the extent to which these results may be related to the specific camp setting.

First, it could be supposed that the lack of any notable psychological benefit following camp participation may be associated with the absence of specific, structured psychological interventions in the camp activities. Given that the camp was not designed to offer psychological treatment to individuals, it is possible that the brief camp experience is not strong enough to be associated with changes in the psychosocial aspects that were examined here. Although diabetes camps may provide an opportunity for recreation and education that can help youths with diabetes better cope with the stresses related to diabetes management, only a structured psychological intervention specifically focused on improving psychosocial functioning may ensure effectiveness. As clearly highlighted in the International Society for Pediatric and Adolescent Diabetes' (ISPAD's) guidelines for psychological care of children and adolescents with T1D (1), an integrated approach to T1D management addressing psychological and physical management of diabetes, both for the youths and their parents, is recommended. Dedicated meetings lead by a psychologist should be included in camp activities and can be held every day, with the aim of discussing various topics, some suggested by the youths, concerning their main issues (e.g., recognizing emotions, daily problems related to T1D management, fears and difficulties of living with T1D); promoting emotional support among campers with similar experiences; and encouraging participants to share their experiences, to give and receive help, and to learn from others' experiences. This intervention could provide the opportunity to bond with others and to share the feelings (such as fear, shame, anger, etc.) that one can experience as a result of having T1D; it could also reinforce the opportunity to form relationships with peers. In addition, to improve long-term camp effects, it could be useful to provide continuous education and psychological intervention through periodical group psychoeducational sessions during clinic visits. Thanks to the support of the multidisciplinary team (diabetologist, psychologist, dietician, and nurse), these group sessions might allow clinicians to monitor and possibly reinforce the skills and knowledge acquired during camp. At the same time, they might also help children and parents to identify attitudes and behaviors that potentially affect good metabolic control, providing further insights for use in planning and organizing diabetes camps.

Second, the lack of camp activities focused on parents' needs may have played a role in the present findings.

It should be noted that other evidence in studies examining parents' diabetes-specific emotional distress after camp (22) and parents' general perceptions of the camp experience (21, 25, 26) overwhelmingly (except 35) came from studies on camps that only included youths as campers. Given how seriously diabetes management impacts parents' lives—as demonstrated by the stress and burden frequently reported in studies on caregivers, who are described as overwhelmed by the demands of their children's T1D (82, 83)—and given the positive association connecting parents' well-being, family dynamics, and children's metabolic control (35), it is difficult to image significant changes in parents' diabetes perceptions after a camp experience without their direct involvement. Parent-oriented activities should be planned alongside camp activities. If the camp experience is conceived to also promote self-care skills and independence in management (especially from parental monitoring), it is possible to promote parent-oriented activities that do not necessarily require the parents to take part in group activities. In order to avoid negatively affecting the self-management experience provided by the camp, these activities should be appropriately scheduled and possibly carried out in a dedicated location.

Finally, an explanation of these results should also consider that the duration of the camp was only 1 week long and that data were collected at only one time point (after 3 months). We do not know whether the length of the camp was too brief to observe significant changes following the experience and/or whether possible changes in diabetes improvement might be identifiable over a longer time period. Collecting data not only before and 3 months after camp but also at several months to years after camp ended might have allowed us to identify potential changes. Additionally, no activities after the camp were organized to continue to reinforce the skills learned during camp. It could be hypothesized that youths, lacking an opportunity to strengthen their new learning, may have lost the skills as soon as they returned home.

Generally, the present findings must be interpreted with caution, due to some methodological limitations of the study. First, the self-selection aspect of the participants' recruitment may have introduced sample selection bias. Additionally, the final sample size was small, and all participants attended the same pediatric diabetology service; thus, it is not representative of the entire population. This limitation affects the generalizability of the findings and the external validity of the study. Furthermore, neither the time of diagnosis nor the age at diagnosis were considered nor a comparison or control group of youths who did not attend camp. Moreover, even though only those who attended the camp for the first time were included in this study, so that their responses could not biased by previous experiences, this study relied on self-report data; therefore, subjective perceptions of behaviors, thoughts, and feelings might not have been sincerely, accurately, or fully revealed.

Further research is necessary to address these limitations and to expand knowledge on the psychological experience of patients and families associated with youths' camp experiences.

Despite these limitations, the present results have important theoretical and practical implications. In its attempt to update and expand the existing literature on the benefits of summer camp experiences for youths with T1D, this study sheds light on the need to further analyze themes for how to make camp an experience that enhances the youth's attitude toward their illness and their confidence in diabetes management. Conflicting literature results on camp efficacy fail to answer the question of which aspects of programs should be altered and which elements make programs successful or unsuccessful. Even though much evidence supports the positive effects of camp experiences, the elements responsible for that success have largely remained unclear, leaving this issue as an unresolved problem. From a practical point of view, the present findings clearly indicate that, in addition to didactic and interactive child-centered education, it is also important to involve a psychologist in order to better structure possible activities. Clinical psychologists should play a role in designing camp curricula and in determining, together with medical team, how the entire camp program/organization might be adapted to meet the needs of campers, potentially according to age groups. Psychologists should also play a role in evaluating camping programs and making the information obtained from such program evaluations available to camp staff and other members of the care team (26).

An important next step in this line of research is longitudinal research assessing whether camp participation is related to long-term improvement outcomes in self-management and in psychosocial functioning, as this will provide valuable information for designing diabetes camp programs.

## Data Availability Statement

The raw data supporting the conclusions of this article will be made available by the authors, without undue reservation.

## Ethics Statement

The studies involving human participants were reviewed and approved by Ethics Commitee of University of Campania “Luigi Vanvitelli”. Written informed consent to participate in this study was provided by the participants' legal guardian/next of kin.

## Author Contributions

AT designed the study, analyzed the data, and wrote the manuscript. DI supervised this work, designed the study, and contributed to the manuscript. CC, AC, and AZ collected data and contributed to the data analyses and to the manuscript. All authors contributed to the article and approved the submitted version.

## Conflict of Interest

The authors declare that the research was conducted in the absence of any commercial or financial relationships that could be construed as a potential conflict of interest. The handling editor declared a past collaboration with the authors DI and AZ.
